# CLEC3A-Derived Antimicrobial Peptides Reduce *Staphylococcus aureus* Bacterial Counts in an In Vivo Biomaterial-Associated Infection Mouse Model

**DOI:** 10.3390/pharmaceutics17020234

**Published:** 2025-02-12

**Authors:** Denise Meinberger, Gabriele Hermes, Bent Brachvogel, Gerhard Sengle, Dzemal Elezagic, Annika Roth, Johannes Ruthard, Thomas Streichert, Andreas R. Klatt

**Affiliations:** 1 Institute for Clinical Chemistry, Medical Faculty and University Hospital Cologne, University of Cologne, 50931 Cologne, Germany; dmeinber@smail.uni-koeln.de (D.M.); gabriele.hermes@uk-koeln.de (G.H.); annika.roth@med.uni-duesseldorf.de (A.R.); johannes.ruthard@uk-koeln.de (J.R.); thomas.streichert@uk-koeln.de (T.S.);; 2 Department of Pediatrics and Adolescent Medicine, Experimental Neonatology, Faculty of Medicine and University Hospital Cologne, University of Cologne, 50931 Cologne, Germany; bbrachvo@uni-koeln.de; 3 Center for Biochemistry, Medical Faculty and University Hospital Cologne, University of Cologne, 50931 Cologne, Germany; gsengle@uni-koeln.de; 4 Department of Pediatrics and Adolescent Medicine, Faculty of Medicine and University Hospital Cologne, University of Cologne, 50931 Cologne, Germany; 5 Center for Molecular Medicine Cologne, University of Cologne, 50931 Cologne, Germany; 6 Cologne Center for Musculoskeletal Biomechanics, 50931 Cologne, Germany

**Keywords:** CLEC3A, C type lectin, antimicrobial peptides, peptide modification, in vivo mouse model, biomaterial-associated infection model

## Abstract

**Background/Objectives:** Biomaterials are an essential part of healthcare for both diagnostic and therapeutic procedures. Although some biomaterials possess antimicrobial properties, introducing biomaterial into the body may lead to infections due to bacterial adhesion on their surfaces and still poses a major clinical problem. Peptides derived from the human cartilage-specific C-type lectin domain family 3 member A (CLEC3A) show a potent antimicrobial effect. In addition, coating titanium, a commonly used prosthetic material, with the CLEC3A-derived AMPs HT-47 and WRK-30 greatly reduces the number of adherent bacteria in vitro. The aim of this study was to evaluate the effectiveness of CLEC3A-derived peptides HT-47 and WRK-30 in reducing bacterial adhesion and mitigating infection in vivo in a murine biomaterial-associated infection model. **Methods**: To do so, an in vivo mouse infection model was used, where titanium plates—either uncoated or coated with chimeric CLEC3A-derived peptides TiBP-HT-47 and TiBP-WRK-30—were implanted subcutaneously into mice. This was followed by the introduction of Staphylococcus aureus bacterial cultures to induce a biomaterial-associated infection. After 24 h, the titanium plates, surrounding tissue, and mice blood samples were investigated. **Results**: CLEC3A-coated titanium plates lead to a significantly lower bacterial count than uncoated ones. Additionally, they prevent the infection from spreading to the surrounding tissue. Moreover, mice with CLEC3A-coated implants display lower IL-6 serum levels and therefore decreased systemic inflammation. **Conclusions**: In conclusion, in this biomaterial-associated infection mouse-model, CLEC3A-derived peptides show in vivo antimicrobial activity by reducing bacterial burden on biomaterial and wound tissue and decreasing systemic inflammation, making them promising candidates for clinical applications.

## 1. Introduction

Millions of arthroplasties are performed every year. An estimated 1–2% of these procedures lead to septic arthritis. Septic arthritis can lead to irreversible loss of joint function and shows high mortality rates. One of the most promising candidates for the treatment of septic arthritis and other infections are antimicrobial peptides (AMPs) [1]. AMPs are short peptides of up to 50 amino acids, often displaying a high positive net charge [2]. AMPs exhibit amphipathic α-helices, β-sheet formations, loops, and extended random coils as secondary structures [3]. They are commonly found as a component of immunity in various species showing potent antimicrobial activity [4,5]. AMPs bind to and incorporate into the bacterial membrane or cell wall, lysing the microbes by membrane destabilization and pore formation [6]. Several antimicrobial peptides, such as LL-37, nisin, or melittin, are in clinical trials [7,8]. A low cytotoxicity on eukaryotic cells and long biostability play a decisive role in their therapeutic application.

**Figure 1:**
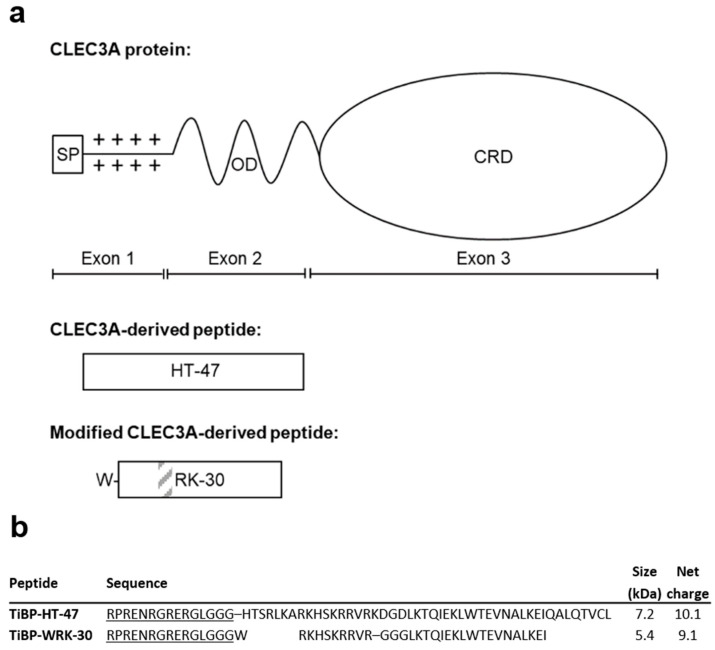
Modified CLEC3A-derived AMPs (**a**). CLEC3A protein structure with signal peptide (SP), oligomerization domain (OD), and carbohydrate recognition domain (CRD). CLEC3A-derived peptides HT-47 aligned to their origin in the CLEC3A protein. The modified CLEC3A-derived peptide WRK-30 is depicted underneath; the grey-striped area indicates the inserted triple glycine linker, and the W in front of the black-framed box indicates the added tryptophan residues. Adapted from Elezagic et al. (2019) and Meinberger et al. (2023) [9,10]. Amino acid sequences of the used peptides TiBP-HT-47 and TiBP-WRK-30 (**b**). TiBP sequence is underlined. Net charge at pH 7 was calculated using the Innovagen Protein Calculator (www.pepcalc.com/protein-calculator, accessed on: 1 February 2025).

Recently, CLEC3A-derived AMP HT-47 and the modified CLEC3A-derived AMP WRK-30 (Figure 1) were identified as promising candidates for clinical applications [9]. HT-47 showed potent antimicrobial activity against septic arthritis-causing bacterial strains. Moreover, when coating titanium, a common prosthetic material, with this peptide, bacterial adhesion was significantly reduced after incubating titanium with bacteria. WRK-30, a modified version of HT-47, can kill *S. aureus* and methicillin-resistant *S. aureus* (*MRSA*) in lower concentrations, showing reduced cytotoxicity compared to its precursor [9,10]. A possible clinical application of HT-47 and WRK-30 could therefore be the prevention of bacterial adhesion to prosthetic materials by coating the AMPs to the biomaterial [11,12], thereby preventing the risk of septic arthritis in joint replacement surgeries and in all other surgeries requiring implanting biomaterials [13]. Coating the biomaterial such as titanium can be achieved by adding a titanium binding protein (TiBP) sequence to the AMPs and thus generating chimeric peptides [14,15].

To date, the antimicrobial activity of at least 3000 peptides has been shown in vitro, but only a few AMPs retain their antimicrobial activity in vivo [16]. Therefore, investigating AMPs’ antimicrobial effect in an animal model is crucial for future therapeutic use. Prosthetic joint implants and investigations in perioperative prosthetic joint infections are limited to larger mammals such as dogs and horses. On the other hand, these are associated with extensive laboratory requirements and costs, as well as ethical and animal rights issues. Studies with experiments on smaller animals such as mice are scarce. In one of the few such studies, a subcutaneous implant infection model was used to test the in vivo antimicrobial activity of an LL-37-derived AMP [17].

This study evaluates the in vivo antimicrobial activity of CLEC3A-derived peptides using a mouse model of biomaterial-associated infection. Specifically, it investigates the peptides’ ability to reduce bacterial load on titanium plates and surrounding tissue. Additionally, the study assesses systemic inflammation by measuring IL-6 concentrations in mouse serum. Through this comprehensive investigation, the study explores the potential of CLEC3A-derived peptides as promising candidates for clinical applications.

## 2. Materials and Methods

### 2.1. Mice

Female C57BL/6N mice were purchased from Charles River Laboratories Germany (Sulzfeld, Germany). Animals were housed in a specific pathogen-free S1 animal facility and kept under temperature and humidity-controlled conditions and a 12 h light and 12 h dark schedule in IVC cages. Food and water were provided ad libitum, and all animals were euthanized humanely using CO_2_. Experiments were performed in accordance with German law, and the procedure was approved by the local government authority LANUV under permit no. 81-02.04.2019.A458. Before including shipped mice in the experiments, they were given an equilibration time of 1 week to settle into their new environment and reduce stress. Mice were subjected to the surgery when they reached the age of 7 weeks. Animals were only taken out of their cages and handled under class II laminar flow biological safety cabinets.

### 2.2. Bacterial Strains

*Staphylococcus aureus* (ATCC-29213) was used and cultivated in tryptic soy broth (TSB) (Merck, Darmstadt, Germany) liquid culture as well as on plates TSB agarose plates. Bacteria were grown at 37 °C under constant agitation in TSB to an OD (620 nm) of 0.5. For further use, the bacterial solution was adjusted to 10^3^ CFU in 20 µL of sterile 0.9% NaCl.

### 2.3. Peptides

Chimeric peptides TiBP-HT-47 and TiBP-WRK-30 were custom ordered from Genosphere Biotechnologies (Paris, France) with a purity of >95% and a salt exchange from TFA to acetate.

### 2.4. Peptide-Coating of Titanium Plates

Titanium plates (Ti-plates) with a thickness of 0.5 mm, a diameter of 4 mm, and a purity of +99.6% were purchased from Goodfellow/Merck (Darmstadt, Germany). Ti-plates were soaked in 70% ethanol for 72 h and afterward sonicated for 15 min at 37 Hz in 1:1 acetone:methanol, isopropyl alcohol, and deionized water. Afterward, Ti-plates were transferred into a sterile 96 well plate and sterilized under UV light (254 nm) over a distance of 60 cm for 15 min on each side. To coat the washed and sterilized Ti-plates, they were incubated with 60 µM of the chimeric peptides in PBS at 37 °C under constant agitation overnight. Control Ti-plates were incubated in PBS only. After incubation, the coated Ti-plates were transferred into sterile reaction tubes and washed with 1 mL of PBS twice.

### 2.5. Biomaterial-Associated Infection Mouse Model

Anesthesia of mice was achieved using ketamine (100 mg/kg) and xylazine (10 mg/kg) in 0.9% NaCl to a maximal concentration of 0.05 mL/10 g. Repeated anesthesia was carried out with 1/2 of the initial dose. The depth of the anesthesia was checked by intertoe reflex. The animals were placed on a heating pad, and eye-and-nose ointment was administered to their eyes. The surgical site (neck) was shaved and washed with 70% ethanol, an incision was made, and a subcutaneous (s.c.) pocket was opened. A chimeric peptide-coated Ti-plate was placed inside the s.c. pocket, and 10^3^ CFU bacteria in 20 µL of sterile 0.9% NaCl was pipetted into the pocket. The skin of the animals was closed with VetBond closure glue, and animals received 0.1 mg/kg (max 0.1 mL/10 g) buprenorphine in 0.9% NaCl s.c. for pain management. Control animals were subjected to the same procedure but receiving an uncoated Ti-plate. The mice woke under supervision, and after the surgery, they were transferred into separate cages. Six h and 21 h after the surgery, the mice received another dose of buprenorphine intraperitoneally, and their water was supplemented with 0.009 mg/mL buprenorphine for access during the whole monitoring phase of 24 h. After 24 h, the mice were euthanized humanely using CO_2_. Blood was drawn via cardiac puncture into EDTA-filled vials, the Ti-plate was taken out, and the tissue surrounding the Ti-plate (excluding the skin) was taken. The blood was centrifuged for 10 min at maximum speed, and the obtained serum was frozen at −80 °C for later use. To harvest the bacteria, a previously described method was modified [18]. Briefly, Ti-plates were taken up in 100 µL of 0.9% NaCl and sonicated for 15 min at 37 Hz, and the resulting solution was streaked out directly as well as in serial dilutions onto pre-warmed TSB agar plates. The dissected tissue was taken up in 0.9% NaCl in 5 times the volume of its weight and homogenized for 1 min at medium speed with an Omni TH Homogenizer. Again, the resulting solution was streaked out directly as well as in serial dilutions onto pre-warmed TSB agar plates. Streaked-out agar plates were incubated for 20 h at 37 °C before pictures were taken and colonies were counted.

### 2.6. IL-6 Immunosorbent Assay

To measure the IL-6 concentration in the collected serum of infected mice, the mouse IL-6 ELISA Kit no. KE10007 from ChromoTek&Proteintech Germany (Planegg Martinsried, Germany) was used. Samples were measured in duplicate, and the assay was performed according to the manufacturer’s instructions and provided protocol.

### 2.7. Statistical Analysis

Prism 9.3.1 (471) (GraphPad, San Diego, CA, USA) was used for statistical analysis of the results. Graphs show calculated averages and standard deviations obtained from four individual experiments (a total of 12 mice were examined). Statistical significance was determined with a paired ANOVA test followed by multiple comparisons comparing TiBP-HT-47 and TiBP-WRK-30 to the uncoated control (Ctrl) and lastly a Dunnett test.

### 2.8. Manuscript Preparation

This manuscript was refined for language clarity and grammar corrections using ChatGPT-4 Turbo and Grammarly (https://app.grammarly.com/, 5 February 2025).

## 3. Results

### 3.1. Analysis of Bacterial Count on the Titanium Plate and in the Wound Tissue

**Figure 2:**
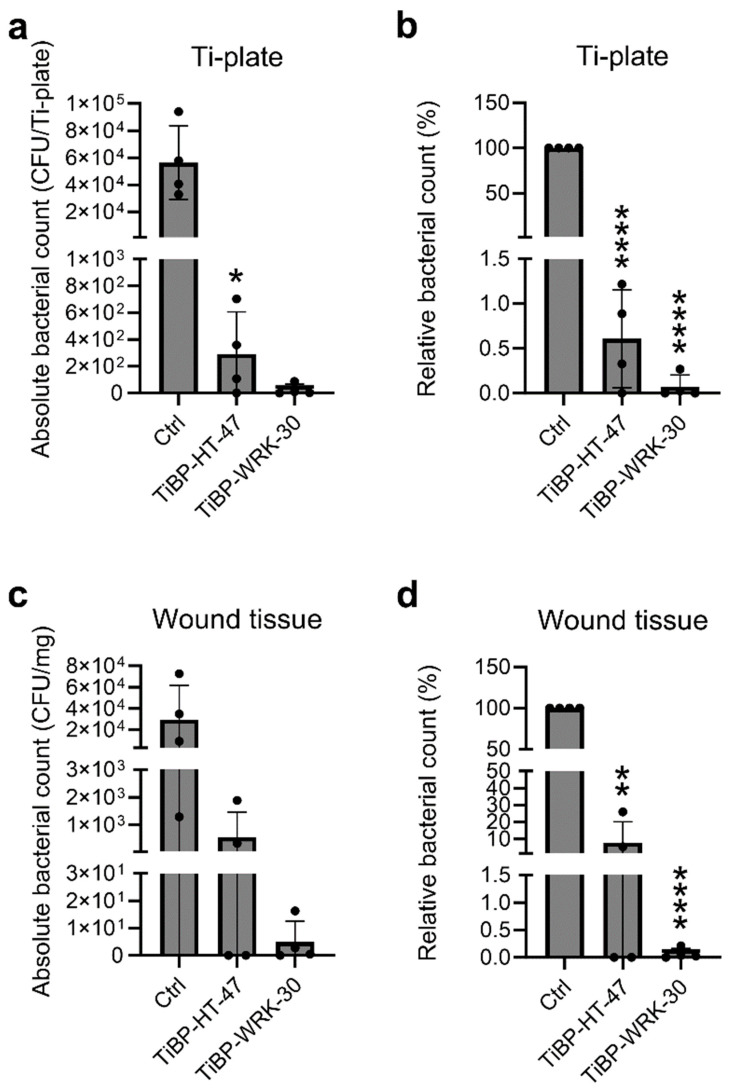
Bacterial burden on the titanium plate (Ti-plate) and in the wound tissue of infected mice. (**a**) Bacterial count in absolute numbers found on the Ti-plates. * *p* = 0.0431. (**b**) Bacterial burden found on the Ti-plates normed to the controls in %. (**c**) Bacterial count in absolute numbers found in the wound tissue. **** *p* < 0.0001; (**d**) Bacterial burden found in the wound tissue normed to the controls in %. ** *p* = 0.0011; Depicted are averages with standard deviations. Statistical significance was calculated by comparing TiBP-HT-47 and TiBP-WRK-30 to the uncoated control (Ctrl). Experiments were repeated four times. The *Y*-axis scaling was adjusted to ensure the bars for HT-47, WRK-30, and their differences were visible.

CLEC3A-derived antimicrobial peptides (AMPs) were coated onto titanium plates by utilizing the binding properties of a titanium-binding peptide (TiBP). This was achieved by creating chimeric peptides that incorporated the TiBP amino acid sequence, which has a strong affinity for titanium surfaces, along with the CLEC3A-derived AMP sequences. The TiBP linker facilitated stable attachment of the antimicrobial peptides to the titanium plates, as described previously [10]. These chimeric peptides were then tested for their in vivo efficacy using a murine biomaterial-associated infection model. To assess their antimicrobial activity, the bacterial count on the implanted Ti-plates was determined. Ti-plates coated with TiBP-HT-47 and TiBP-WRK-30 showed a significantly reduced number of viable colonies compared to the uncoated control plates (Figure 2a,b). On average, coating the Ti-plates with TiBP-HT-47 led to a 99.4% reduction in the bacterial count (confidence interval ±0.89) and with TiBP-WRK-30, a 99.9% reduction (confidence interval ±0.19) (Figure 2b). Thus, TiBP-HT-47 and TiBP-WRK-30 not only retain their antimicrobial activity when immobilized on a surface but also demonstrate effectiveness in an in vivo setting, making them promising candidates for potential therapeutic applications.

Infections after implantation do not take place only as biofilms on the implanted biomaterial but are also able to spread into the surrounding tissue. Therefore, the bacterial burden in the wound tissue surrounding the implanted Ti-plate was quantified. A reduction in viable bacteria in the wound tissue was observed (Figure 2c,d). On average, TiBP-HT-47 achieved a reduction of 89.3% (confidence interval ±19.62) and TiBP-WRK-30 once more of 99.9% (confidence interval ±0.16) (Figure 2d). This shows that TiBP-HT-47 and TiBP-WRK-30 not only reduce the bacterial count on the biomaterial they are bound to but also in the surrounding wound tissue.

### 3.2. Analysis of IL-6 Concentration in the Mouse Serum

**Figure 3:**
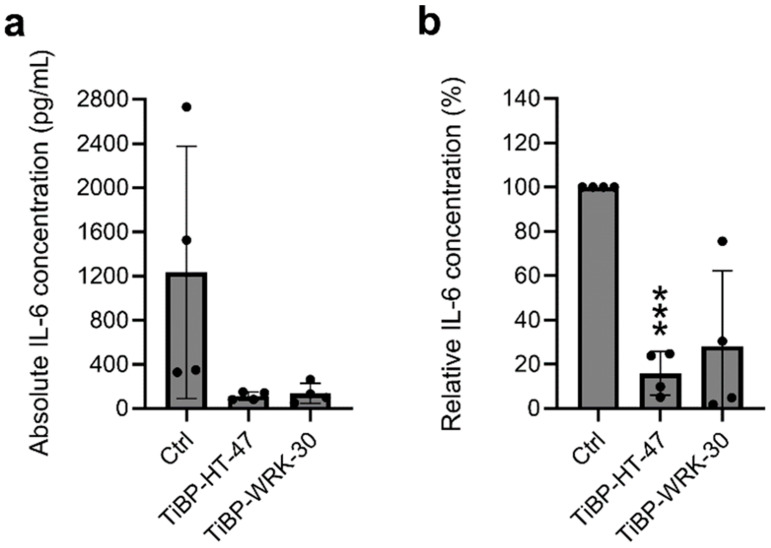
IL-6 concentration in the serum of infected mice was measured by immunosorbent assay. (**a**) Absolute IL-6 concentration in pg/mL. (**b**) IL-6 concentration normed to the control in %. *** *p* = 0.0007. Depicted are averages with standard deviations. Statistical significance was calculated by comparing TiBP-HT-47 and TiBP-WRK-30 to the uncoated control (Ctrl). Single measurements were performed in duplicates and experiments in quadruplicates.

IL-6 was used as a biomarker for systemic inflammation. IL-6 is a pro-inflammatory cytokine released by macrophages and fibroblasts upon a bacterial infection [19]. The IL-6 concentration in the serum of the mice after 24 h of infection was measured to assess the severity of the infection. Excitingly, the serum concentration of IL 6 in the TiBP-HT-47 and TiBP-WRK-30 treated mice was reduced (Figure 3). On average, TiBP-HT-47 led to a reduction of 84.2% (confidence interval ±15.67), and TiBP-WRK-30 of 71.9% (confidence interval ±54.24) (Figure 3b).

## 4. Discussion

Perioperative infection is a serious complication in joint replacement surgery. Other biomaterial-associated infections, such as those affecting catheters or dental implants, are relatively common and carry significant medical and economic consequences. Current strategies to reduce biomaterial-associated infections involve the development of anti-adhesive surfaces, tissue-integrating surfaces, contact-killing surfaces, and surfaces that incorporate and release antimicrobials. These approaches show promise in mitigating the risk of infections and improving patient outcomes [20]. A particularly promising strategy is the coating of biomaterials with antimicrobial peptides (AMPs). AMPs exhibit broad-spectrum activity against a wide variety of pathogens and offer a low risk of resistance development due to their rapid, cell-death-inducing antimicrobial mechanism. This makes them an attractive option for combating infections associated with medical implants while minimizing the challenges posed by antimicrobial resistance [4].

Two major challenges still hinder the therapeutic potential of AMPs: maintaining their antimicrobial activity in vivo and selecting an appropriate animal model. Animal models are crucial for orthopedic research, as they enable the testing of interventions that cannot be replicated in vitro. Different animal models serve distinct purposes: large animal models are more suitable for testing human-scale, multidisciplinary treatment strategies, while small animal models are particularly valuable for early studies on pathophysiology and proof-of-concept research, offering the added benefit of lower study costs [21]. Therefore, we designed a murine, biomaterial-associated infection model, in which uncoated or AMP-coated titanium plates are implanted subcutaneously in the neck of the mice, and bacteria are simultaneously introduced into the wound. Coating the titanium plates with CLEC3A-derived AMPs lead to a significant decrease in bacteria adhering to the plates.

Another critical aspect of biomaterial-associated infections is the bacterial colonization of the tissue surrounding the implant. This colonization typically occurs due to a compromised local immune response, which is exacerbated by the presence of both bacteria and the foreign body [22]. In cases of re-implantation, these bacteria can lead to persistent infections that are extremely difficult to eradicate. Patients whose infected biomaterials must be removed often require extended antibiotic treatment before re-implantation can take place, resulting in prolonged patient suffering, extended hospital stays, and increased healthcare costs [23]. Consistent with other in vivo studies of biomaterial-associated infections [24,25,26], we also observed bacterial infiltration in the surrounding tissue. However, in addition to the reduction of bacterial load on the biomaterial surface, coating the biomaterial with CLEC3A-derived antimicrobial AMPs also significantly reduced bacterial load in the surrounding tissue.

The initial adhesion of bacteria to the biomaterial surface marks the first step in biofilm formation, which can become established and persist not only on the biomaterial surface but also on surrounding host tissues. Biofilms provide a protective environment for bacteria, creating a significant barrier to both the host immune response and antibiotic treatments [27]. One of the few studies investigating the timeframe of *S. aureus* biofilm formation demonstrated that, after 24 h of incubation, 34.6% of isolates were capable of forming biofilms. This percentage increased to 80.8% after 72 h [28]. While studies using longer infection durations are necessary for the clinical translation of biomaterial coatings, the 24-h duration of our model was sufficient to induce bacterial colonization on the tissue and trigger systemic inflammation. As such, this model serves as an initial proof of concept for assessing the in vivo activity of CLEC3A-derived AMPs.

Typically, during planktonic bacterial infections, macrophages are triggered via the classical pathways of toll-like receptors 2 (TLR-2) and 9 (TLR-9). This activation leads to the phagocytosis of bacteria and the production of pro-inflammatory mediators such as iNOS, TNF-α, IL-1β, IFN-γ, and IL-6 [29]. An excessive inflammatory reaction is therefore a common complication of spreading infections. In our mouse model, serum IL-6 concentrations were significantly lower in mice implanted with AMP-coated titanium plates compared to those with uncoated plates. This reduction is likely attributable to the decreased bacterial loads on the coated plates and in the surrounding tissue. However, an inconsistency was observed with WRK-30-coated Ti-plates, which showed higher cytokine levels despite lower bacterial accumulation. This discrepancy may be attributed to the small sample size used in the study and the physiological variation among individual mice. Additionally, the surgical procedures themselves may have introduced variability in cytokine levels. Further studies with larger sample sizes are needed to clarify these findings and validate the results.

In addition, there is a possibility that CLEC3A-derived peptides interact with the immune system by directly inducing the production of anti-inflammatory cytokines, a phenomenon observed with several other well-known AMPs, such as human beta-defensin 1 or LL-37 [30]. It is important to note, however, that staphylococcal biofilm formation itself can also modulate the host immune response, skewing it toward an anti-inflammatory state by altering macrophage phenotypes [31]. To better understand the immune mechanisms in implant-associated infections, further studies incorporating comprehensive cytokine, chemokine, and cell population analyses are essential.

Titanium, while renowned for its biocompatibility, high strength-to-weight ratio, and corrosion resistance, does pose risks such as wear that can release metal ions into the body, potentially causing adverse reactions. However, titanium remains the material of choice for orthopedic implants due to its superior integration with bone tissue and advancements in surface modifications and manufacturing techniques. Ongoing research, including the use of machine learning for optimizing titanium alloys, further enhances its performance and longevity in medical applications [32]. The use of Ti-binding peptides for coating Titanium is by far not the only method used to get AMPs on its surface. Other well-described methods include surface attachment through a cysteine residue [33], using nanotubes for a controlled release [34], or click chemistry using Cu-ions [35]. A limitation of our study is the absence of characterization data for the titanium plates and future studies with surface characterization would clarify binding stability and coating integrity in vivo. Nevertheless, a large body of literature supports the stable binding of TiBP sequences and chimeric peptides containing this sequence to titanium surfaces [14,15,17].

## 5. Conclusions

In conclusion, CLEC3A-derived peptides HT-47 and WRK-30 retain their antimicrobial activity in vivo in a murine biomaterial-associated infection model. On average, TiBP-HT-47 achieved a reduction of 89.3% (confidence interval ±19.62), and TiBP-WRK-30 achieved a reduction of 99.9% (confidence interval ±0.16) in bacterial counts. Regarding IL-6 concentrations, TiBP-HT-47 led to a reduction of 84.2% (confidence interval ±15.67), while TiBP-WRK-30 resulted in a reduction of 71.9% (confidence interval ±54.24). Both AMPs not only reduce the bacterial count on the implanted biomaterial and in the wound tissue but also limit systemic inflammation, making them promising candidates for therapeutic applications.

## 6. Patents

Antimicrobial CLEC3A peptide fragments (AMP) are covered by a pending German patent (DE 10 2018 113 988.8 A1) and an international patent application (PCT/EP2024/053920).

## Data Availability

The original contributions presented in the study are included in the article material, further inquiries can be directed to the corresponding author.
